# Potential Alternative Strategy against Drug Resistant Tuberculosis: A Proteomics Prospect

**DOI:** 10.3390/proteomes6020026

**Published:** 2018-05-28

**Authors:** Divakar Sharma, Deepa Bisht, Asad U. Khan

**Affiliations:** 1 Department of Biochemistry, National JALMA Institute for Leprosy and Other Mycobacterial Diseases, Tajganj, Agra 282004, India; 2 Medical Microbiology and Molecular Biology Laboratory, Interdisciplinary Biotechnology Unit, Aligarh Muslim University, Aligarh 202002, India

**Keywords:** tuberculosis, infectious diseases, drug resistance, proteomics, alternative strategies

## Abstract

*Mycobacterium tuberculosis* is one of the deadliest human pathogen of the tuberculosis diseases. Drug resistance leads to emergence of multidrug-resistant and extremely drug resistant strains of *M. tuberculosis*. Apart from principal targets of resistance, many explanations have been proposed for drug resistance but some resistance mechanisms are still unknown. Recently approved line probe assay (LPA) diagnostics for detecting the resistance to first and second line drugs are unable to diagnose the drug resistance in *M. tuberculosis* isolates which do not have the mutations in particular genes responsible for resistance. Proteomics and bioinformatic tools emerged as direct approaches for identification and characterization of novel proteins which are directly and indirectly involved in drug resistance that could be used as potential targets in future. In future, these novel targets might reveal new mechanism of resistance and can be used in diagnostics or as drug targets.

## 1. Introduction

### 1.1. Current Scenario

Tuberculosis (TB) is one of the deadliest communicable infectious diseases globally and was one of the top ten causes of death worldwide in 2015 [1]. The WHO Global TB Report, (2016) reported 10.4 million new (incident) TB cases worldwide, of which 5.9 million (56%) were among men, 3.5 million (34%) among women and 1.0 million (10%) among children [1]. People living with HIV accounted for 1.2 million (11%) of all new TB cases. There were an estimated 1.8 million TB deaths, among them 0.4 million deaths resulted from TB disease among people living with HIV [1]. In 2015, there were an estimated 480,000 new cases of multidrug-resistant TB (MDR-TB) and an additional 100,000 people with rifampicin-resistant TB (RR-TB) who were also newly eligible for MDR-TB treatment [1]. The six countries that stood out as having the largest numbers of incident cases in 2015 were (in descending order) India, Indonesia, China, Nigeria, Pakistan and South Africa (combined, 60% of the global). Of these, China, India and Indonesia alone accounted for 45% of global cases. In the world of tuberculosis, India accounts for a significant number and is home to more than 25% of all TB cases and deaths worldwide [1]. India is the second-most populous country in the world with one fourth of the global incident TB cases annually. According to the Revised National TB Control Programme RNTCP-TB report of 2016, India had 2.2% and 15% new and retreated MDR cases, respectively [2]. Mortality attributed to infection of *Mycobacterium tuberculosis* has been devastating and is continually increasing within certain countries. India is a signatory to the World Health Assembly which has endorsed sustainable development of goals and global ‘End TB Strategy’ that calls for a world free of tuberculosis, with measurable aims of 50% and 75% reduction in incidence and related deaths, respectively, by 2025, and corresponding reductions of 90% and 95% by 2035, as well as zero catastrophic expenditure due to TB [3]. The latest treatment outcome data show a treatment success rate of 83% for TB (2014 cohort), 52% for MDR-TB (2013 cohort) and 28% for extensively drug-resistant TB (XDR-TB; 2013 cohort) [1].

### 1.2. Cumulative Effort of Government Bodies and Public–Private Consortia

Worldwide, MDR- and XDR-TB are transmitted and thousands of patients languish on waiting lists to receive effective treatment. Yet, the public understanding of TB is limited and conversations about TB research and their implementation often remain isolated. It is important to convey to a wider audience that our current tools for prevention, diagnosis and treatment are not enough. In India, synergistic efforts of stakeholders involved in TB control are the key towards realizing the goal of “Universal access to TB care and treatment for all”. The RNTCP has partnerships with the Indian Medical Association (IMA), Catholic Bishops’ Conference of India (CBCI), Foundation for Innovative New Diagnostics (FIND) and The UNION and World Vision. These partners strengthen the engagement of communities and community systems, increase the fast diagnosis for drug resistance TB and have complemented RNTCP’s efforts towards universal access to TB care. Now, it can show that public–private consortia may also play an important role in TB diagnostics and drug discovery which could prevent MDR-TB and XDR-TB. India and other so-called third world countries majorly contribute to TB prevalence as well as resistance globally; therefore, the world scenario has been drastically changed and these countries have become the focus of more attention. The reason behind this worsening situation is not only poverty and unawareness of TB, but rather scarcity of current tools for TB prevention, diagnostics and treatment which need to be strengthened in order to control the deadly situation that are needed in order to achieve the goal of TB elimination by 2050.Most recently, the National JALMA Institute for Leprosy and Other Mycobacterial Diseases (ICMR) launched a mobile TB diagnostic van for targeted intervention to expand and strengthen TB control in tribal populations under RNTCP.

### 1.3. Slow Progress in Vaccines, Diagnostics and Drug Discovery

Over the past 50 years, the *Mycobacterium bovis* bacille Calmette Guérin (BCG) vaccine against TB has maintained its position as the world's most widely used vaccine and showed highly variable efficacy (0–80%) in different trials across the world [4]. It significantly reduced the risk of tuberculosis by an average of 50%. There are more than ten clinical candidates in various phases of clinical trials, which include protein or adjuvant vaccines, viral-vectored vaccines, mycobacterial whole-cell vaccines, recombinant live and an attenuated *Mycobacterium tuberculosis* vaccine [5]. Sputum smear microscopy is the most common TB diagnostic tool and culture remains the gold standard across the globe. Use of rapid molecular testing such as the line probe assay (LPA) is increasing for detection of multidrug-resistant *M. tuberculosis* strains, and recently, the Revised National TB Control Programme (RNTCP) has stated in its Annual Status report “TB India 2016” that The National Committee of Operational Research has approved a study for the validation of a second line LPA for detecting resistance to fluoroquinolones, aminoglycosides (kanamycin, amikacin) and cyclic peptides (Capreomycin) [2]. Chemotherapy has reduced the incidence of TB caused by susceptible strains, but the circumstances are not the same in case of MDR-TB and XDR-TB. Generally, physicians do not support the use of drugs to which TB bacilli are resistant, and on the other hand, first and second line drugs are limited and bacterial responses vary depending on the level of resistance. Widespread development of MDR-TB and its spread has worsened the situation further and, to combat this situation, second-line anti-TB drugs (aminoglycosides and fluoroquinolones) are used. Failure of these second-line anti-TB treatments led to resistance and emergence of the XDR-TB. Sharma et al. (2017) recently suggested that bactericidal and synergistic effects of repurposed/revived drugs along with the latest drugs bedaquiline and delamanid used in the treatment of MDR-TB, XDR-TB and TDR-TB, might be the choice for future promising combinatorial chemotherapy against these bacteria [6]. Current therapies do not offer complete protection against MDR-TB and XDR-TB. There is a need to address this challenge so as to fulfill demand, and there is a need to introduce new drugs for MDR-TB and XDR-TB.

So far, no vaccines have crossed the last trial required for approval; hence, we still depend on the BCG vaccine. Recently approved anti-TB drugs (bedaquiline and delamanid) can potentially induce arrhythmia and are recommended to patients with MDR-TB, XDR-TB and TDR-TB in cases where other options fail. We are still depending on current anti-TB drugs. Recently approved second-line LPA diagnostics for detecting resistance to fluoroquinolones, aminoglycosides (kanamycin, amikacin) and cyclic peptides (capreomycin) are unable to diagnose *M. tuberculosis* which does not have mutations in particular genes. A proteomics approach might be the only possible way for the quick diagnosis of resistance in these strains. The goal of elimination of TB by 2050 will not be achieved without a new effective vaccine, rapid diagnostics for drug resistance and new anti-TB drugs against MDR, XDR-TB and TDR-TB.

### 1.4. Causes of Drug Resistance

Generally, first and second line drug resistance were contributed by mutations in target genes (*KatG*, *InhA*, *ahpC*, *rpoB*, *embB*, *pncA*, *gyrA*, *gyrB*, *rrs*, *rpsL* and *gidB*) of approximately 36–95% *M. tuberculosis* clinical isolates. However, the remaining 5–64% of *M. tuberculosis* clinical isolates do not have these mutations and thus, signify the contribution of some other resistance mechanism(s). *M. tuberculosis* can resist antibiotic actions by the following mechanisms: mutations in target genes [7], modifications of antibiotic molecules through enzymes [8], over expression of novel efflux pumps and porin alterations in the cell wall [9], trapping of drugs and the over expression of proteins involved in neutralizing the effect of drug [10,11,12,13,14,15,16,17].

### 1.5. Alternative Strategies for Drug Resistance Tuberculosis

Repurposing of the drugs (drugs which were used to treat other diseases but are now used against dru- resistant tuberculosis) is one of the novel strategies to resolve the problem of the global emergency of drug resistance [6,18,19]. Host-directed therapy (HDT) is the other possible strategy for treatment of drug-resistant tuberculosis infections such as MDR-TB and XDR-TB [20,21]. Vitamin D supplementation and vaccinations through environmental mycobacteria vaccines (*Mycobacterium indicus pranii*) have also shown promising results against drug-resistant tuberculosis [22,23].

## 2. Proteomics and Bioinformatics Explored Potential Drug Targets and Newer Diagnostic Strategies against Drug-Resistant TB: A Future Perspective

**Figure 1:**
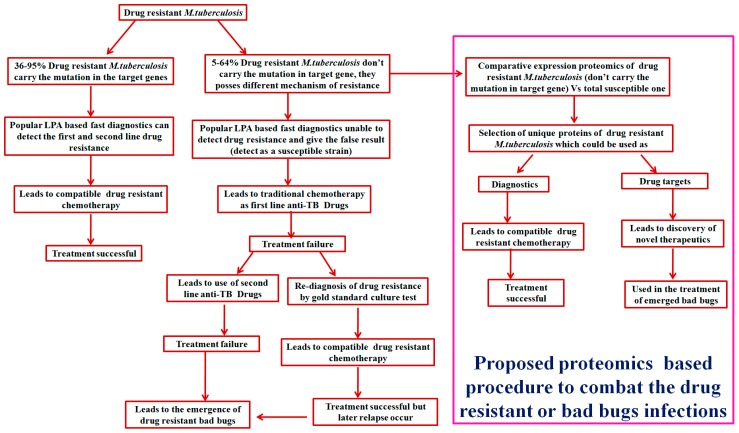
Schematic representation of a proteomics-based approach to combat drug-resistant infections.

Advancement in proteomics coupled with bioinformatics has cleared the doubts regarding drug resistance in *M. tuberculosis* clinical isolates which does not have mutations in the responsible gene and could explore unknown mechanism(s) of resistance. As we know, proteins manifest most biological processes and are attractive targets for developing new drugs and diagnosis of resistance (Figure 1). Expression proteomics {2-dimensional gel electrophoresis (2-DE)} coupled with MALDI-TOF-MS and bioinformatic tools has emerged as a direct approach for the separation, identification and characterization of proteins which could be used to find out potential drug targets and diagnosis of resistance as well as pathogenesis [11,12,13,14,15,16,17,24,25,26,27,28,29,30,31,32,33]. Since the last decade, a panel of discovery (expression), targeted proteomics and bioinformatics-based studies [11,12,13,14,15,16,17,24,25,26,27,28,29,30,31,32,33] have been accumulated on *M. tuberculosis* pathogenesis and drug resistance which showed differential expression of a panel of known and unknown proteins and suggested that these proteins might be used in diagnostics and as drug targets against drug resistant TB. We have previously reported that Rv0148 (hypothetical protein/putative short-chain type dehydrogenase/reductase) was overexpressed in aminoglycosides-resistant *M. tuberculosis*. Rv0148 had an SDR domain; aminoglycosides bind to SDR domains and might hence neutralize the aminoglycosides effect [13]. Furthermore, we found that overexpression of Rv0148 is involved in the shift in MIC of aminoglycosides in recombinant *E. coli* (BL21) [24]. Most recently, we have reported in-silico protein–protein interactions of Rv0148 and suggested that Rv0148, its predictive interactive protein partners and their pathways, such as lipid metabolism, as well as intermediary metabolism and respiration, cumulatively open the secrecy of aminoglycosides resistance in *M. tuberculosis* [33]. In our previous studies of differential expression proteomics, we have discovered that bacterioferritin (Rv1876) and ferritin (Rv3841) were overexpressed in aminoglycosides (amikacin and kanamycin)-resistant *M. tuberculosis* clinical isolates [12,16]. Inducible over-expression of recombinant ferritin in *E. coli* (BL21) increased the MIC shift of AK and KM and made the bacteria more resistant against aminoglycosides drugs [25]. Molecular docking revealed that aminoglycosides drugs (AK and KM) can bind to a bacterioferritin domain of Rv1876 and the ferritin domain of Rv3841 and suggested that overexpression of these proteins might be to neutralize/modulate the aminoglycosides effect and could play probable roles in conferring resistance. Interactome and pupylome analyses of bacterioferritin and ferritin and their interactive partners {hypothetical protein (Rv1877), ferrochelatase, trigger factor and others} suggested that these proteins might be involved in various stress and aminoglycosides drug resistance [27]. Further detailed and in-depth investigations of these interactome and pupylome results could explore aminoglycosides resistance and might be used as potential drug targets against pathogenesis and resistance. These findings might be used in the development of newer therapeutic agents or molecular markers which can directly be targeted to a gene/protein responsible for resistance. Detailed bioinformatic analysis (molecular docking, pupylation and protein–protein interaction) of these proteins might identify pathways and new targets of resistance of *M. tuberculosis* system biology.

Liquid chromatography coupled with tandem mass spectrometry (LC-MS/MS) is another means of proteomics approaches for characterizing the proteomes of different conditions and biological samples at a particular state or time-point. This approach is mainly used for the identification and quantification of proteomes. Apart from that, LC-MS/MS has also been used for the identification of phosphoproteomes, other post-translational modifications (PTMs), conformations and interactions [34]. Exploration of proteomes by LC-MS/MS is based on a bottoms-up proteomics approach is the dominant paradigm for proteomics applications where proteins are firstly digested by a protease like trypsin then peptides are identified and mapped back to proteins for both identification and quantification [35]. For two decades, bottoms-up proteomics approach has dominated through discovery and targeted proteomics [36].

Proteomic profiling of *M. tuberculosis* clinical (susceptible and drug-resistant) strains has not only increased the depth of proteome knowledge about the pathogenesis but also provided information of the resistome which suggested the probable involvement of identified proteins and their associated pathway categories (such as lipid metabolism, virulence detoxification and adaptation, intermediary metabolism and respiration, cell wall and cell processes, PE/PPE, information pathways and regulatory proteins categories, etc.) in drug resistance and mycobacterial pathogenesis. Peters et al. (2016) suggested that LC-MS/MS along with a novel bioinformatics strategy yielded high protein coverage at a high confidence level. Through this approach, the identification of proteins was claimed to reach 80% of the theoretical proteome [37]. They observed and quantified the differential expression of 23 proteins via selected reaction monitoring (SRM) assays and compared that to gene expression data which suggested their relevance to virulence and pathogenesis. These differences may contribute to a *M. tuberculosis* strain’s capacity for surviving within the host, treatment to drug resistance and its spread. Comparative proteomic analysis of H37Rv and H37Ra (virulent and avirulent strains of *M. tuberculosis*) reported that Rv2586c/SecF, Rv0732 (SecY) and Rv2587c (SecD) were 5–6-fold overexpressed. These are part of the general secretory (Sec) pathway which is composed of a membrane-spanning translocation channel composed of various integral membrane proteins. This suggested that the translocation of precursor proteins, which have the ability to bind membrane phospholipids moieties via Sec pathways, contributed to *M. tuberculosis* pathogenesis and resistance. More than a five-fold overexpression of Rv0933, Rv1273c and Rv1819c (transmembrane ATP-binding ABC transporter proteins) may transport various molecules (proteins, peptides, antibiotics, polysaccharides) via transporter systems which could significantly contribute to virulence, pathogenesis of the bacilli and drug resistance [38]. Another study related to the membrane-associated proteins of H37Rv and BCG revealed the significance of membrane proteins in tuberculosis and suggested EsxA as a potential virulence factor. MmpL4, Rv1269c, Rv3137 and SseA proteins are differentially expressed in Beijing strains which suggested that the increased expression of these proteins is associated to the virulence and pathogenesis of Beijing strains [39]. In another study, decreased levels of the protein SseA in the Beijing strains most likely resulted in increased DNA oxidation damage which suggests a higher rate of mutation and accelerated acquisition of drug resistance in these strains [40]. Our group also explored the proteome of ofloxacin-resistant strains and identified fourteen proteins that were up-regulated as compared to ofloxacin-susceptible strains [14]. Major finding of this study were the overexpression of various enzymes involved in metabolic processes (fructose-bisphosphate aldolase, putative CoA-transferase subunit alpha, glyceraldehyde-3-phosphate dehydrogenase, KasA, adenylate kinase and aconitate hydratase) and proteins involved in the translation machinery (EF–Tu, EF–P and ribosome recycling factor) which could be crucial for the pathogenesis under prolonged adverse conditions and drug resistance [14]. Among them, four proteins were proteins with unknown functions (Rv2744c, Rv2140c, Rv3551 and Rv0148) and further molecular docking analysis of these proteins elucidated motifs and domains which could interact with ofloxacin [14]. Another study showed that the abundance of several proteins responsible for the maintenance of the cell-envelope during thioridazine exposure which suggested that thioridazine may increase the cell-envelope permeability for facilitating components uptake [41].

For quantitative measurements of the proteome by LC-MS/MS (DIA/SWATH-MS), a *M. tuberculosis* proteome spectral library has been generated, validated and made publicly accessible [42]. The library contains about 97% of *M. tuberculosis* annotated proteins and has lined the way to study the mycobacterial proteome under various conditions like drug stress or drug resistance. On the basis of shotgun-MS and deep RNASeq, we believe that 3488 proteins are expressed in *M. tuberculosis.* Among these, most of the proteins (70–80%) belong to intermediary metabolism and respiration, virulence detoxification adaptation, lipid metabolism and information pathways which could be involved in *M. tuberculosis* pathogenesis and resistance. A panel of proteins of unknown function/hypothetical proteins or unannotated proteins has been identified by MS-based proteomic approaches which emphasizes that the *M. tuberculosis* genome re-annotation needs to be further refined [42].

Absolute quantification of *M. tuberculosis* proteins and their reorganization after exposure to hypoxia were determined. This showed that proteins associated to the ribosomal machinery remain largely unchanged. Proteins related to DevR/DosR regulon were strongly induced and constituted about 20% of the cellular protein at the time of dormancy whereas the remaining 80% belonged to metabolic processes [43]. Drug-resistant strains also behave like dormant bacteria, so absolute quantification of the proteome of resistant *M. tuberculosis* may help unravel the mystery of drug resistance. Further research on this track could identify a novel way of drug resistance, future diagnostics and drug targets so that the worsening situation of MDR-TB and XDR-TB could be tackled. Such information could be helpful for the development of newer diagnostics and drug targets against drug resistance so that deadly resistance situations could be overcome. Apart from the in vitro research of the pathogen (*M. tuberculosis*), analysis of the host proteome in the relevant environment should be an essential and important goal in the field of infectious diseases. Infected cell lines or primary cells and mouse models can be considered as intermediates towards the analysis of clinical human tissue samples. Our group has also reported eight intraphagosomal expressed proteins of the BCG strain during infection with macrophages [44] which may provide enhanced characterization of MTBC and host-derived targets to the better improvement of TB control. The proteomic approaches described above support the analysis of more complex samples such as bacterial and host components; however, absolute quantifying the pathogen’s proteome during infection will be a daunting task due to the presence of bacterial proteins in less abundance as compared to the host proteome in a complex mixture. These approaches would help to quantify pathogen as well as host proteomes in order to address the issues of host–pathogen interactions.

## 3. Conclusions and Future Perspective

In jest, drug-resistant tuberculosis has worsened the current scenario which has led to the emergence of multidrug-resistant and extremely drug-resistant strains of *M. tuberculosis*. Slow progress in vaccines, resistance diagnostics, treatment and drug discovery increased the global burden of resistance in the current scenario. Apart from a genomic approach (which usually explores mutation-based resistance in principal targets but is unable to detect resistance in which mutations are not observed) proteomics-based approaches (two dimensional gel electrophoresis followed by MALDI-TOF mass spectrometry and LC-MS/MS) coupled with bioinformatics have emerged for the identification and characterization of novel proteins which could be involved in resistance and can be used as potential targets or diagnostics of drug-resistant strains. In the future, these novel targets might identify new mechanisms of resistance and could be used as potential drug targets and in the diagnosis of resistance.
